# Ileocolonic anastomosis–comparison of different surgical techniques: A single-center study

**DOI:** 10.1097/MD.0000000000031582

**Published:** 2022-12-23

**Authors:** Joanna Machowicz, Maciej Wołkowski, Beata Jabłońska, Sławomir Mrowiec

**Affiliations:** a Student Scientific Society, Department of Gastrointestinal Surgery, Medical University of Silesia, Katowice, Poland; b Department of Gastrointestinal Surgery, Medical University of Silesia, Katowice, Poland.

**Keywords:** adenocarcinoma, ileocolonic anastomosis, postoperative complications, right hemicolectomy

## Abstract

Right hemicolectomy (RH) is a common procedure for both benign and malignant colic disease. Different anastomotic types are performed during this procedure. To assess the association between anastomotic type and postoperative complications (PC) in patients undergoing RH. Retrospective analysis of medical records of 72 patients (39 female and 33 male), aged 24 to 93, undergoing open RH in the Department of Gastrointestinal Surgery. Data regarding anastomotic type [end-to-end anastomosis, side-to-side (SSA), end-to-side anastomosis, and side-to-end anastomosis (SEA)], and different clinical factors were collected. There were 21 (29%) end-to-end anastomosis, 25 (35%) SSA, 15 (21%) end-to-side anastomosis, and 11 (15%) SEA in the analyzed group. Adenocarcinoma G2 was the most frequent indication for RH - 30 (42%). Total duration of hospitalization (in days) was the longest (14, 26) after SEA and the shortest (12, 68) after SSA. PC were noted in 17(24%) patients. Wound infection was the most common complication noted in 15(21%) patients. The overall anastomotic leak rate was 7% (5/72). PC were the most frequent after SEA noted in 64% (7/11) including abdominal bleeding and bowel perforation. The overall reoperations rate was 6% (4/72). The overall mortality rate was 4% (3/72). SEA was associated with the highest incidence of postoperative complication however based on this and other studies there are no satisfying conclusions regarding the best choice of anastomosis.

## 1. Introduction

Right sided hemicolectomy (RH) is a commonly performed procedure for both benign and malignant colic disease.^[1]^ One of the crucial skills of a general surgeon is performing anastomosis after colectomy.^[2]^ The anastomosis type selected for colectomy depends on site of the disease, bowel diameter and surgeon’s personal experience.^[3]^ There are different types of ileocolonic anastomosis such as end-to-end anastomosis (EEA), side-to-side anastomosis (SSA), end-to-side anastomosis (ESA) and side-to-end anastomosis (SEA).^[4,5]^ Due to disparity in diameters of colon and ileum, postoperative anastomotic complications are quite common.^[6]^ Choice of the optimum variant of the anastomosis in RH depends on the understanding of pathophysiological consequences of either intestinal anastomosis. EEA seems to be the quickest to fulfill, less traumatic and safe.^[7]^

There are many studies comparing open and laparoscopic colorectal resections^[8–10]^ as well as stapled and handsewn anastomoses in the literature.^[11–13]^ However, there is no study that compares specifically all mentioned types of anastomosis: EEA, SSA, ESA and SEA following right hemicolectomy. Knowledge on the optimal anastomosis configuration following RH is very important for a surgeon.

The aim of this study was to assess association between anastomotic type and postoperative complications in patients undergoing RH.

## 2. Materials and Methods

Seventy two patients who underwent the open procedure of RH between 1st of January 2016 and 31st December of 2018 were included in the study (39 female and 33 male aged 24–93). Their medical records were collected and analyzed retrospectively. All operations were performed in the Department of Gastrointestinal Surgery by experienced surgeons. The choice of anastomotic type was left to a surgeon based on their experience and a clinical picture of the patient. Seventeen patients who underwent laparoscopic procedures were excluded from this study in order to maintain the compared groups homogeneous in terms of surgical approach.

Patient-related factors that were recorded were age and sex. Operation-related factors that were recorded were operating time, blood loss, the number of suture layers, types of sutures, postoperative complications, the number of reoperations and mortality rate. Other data regarding total length of hospital stay, time of hospitalization after the surgery and types of histopathological diagnoses were also collected.

**Figure 1.**
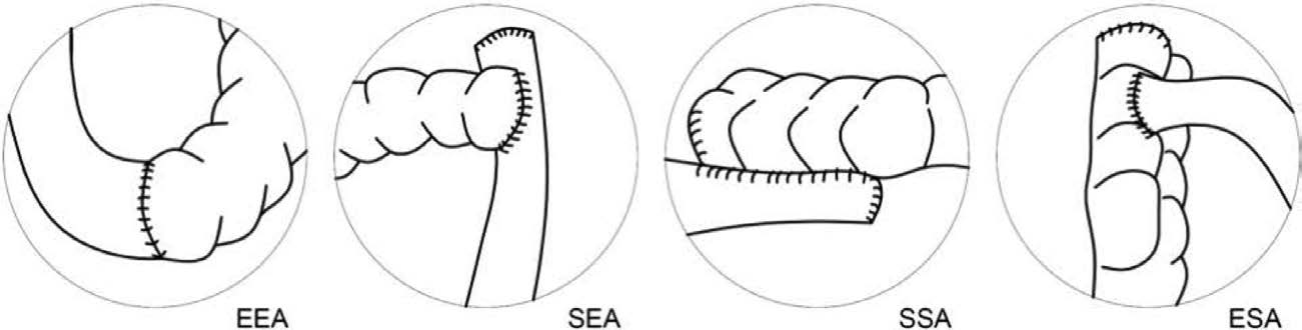
Types of anastomosis. EEA = end-to-end anastomosis, SEA = side-to-end anastomosis, SSA = side-to-side anastomosis, ESA = end-to-side anastomosis.

Patients were divided into 4 groups based on the type of anastomosis performed: EEA, side-to-side (SSA), ESA or SEA. The types of anastomosis are presented in Figure 1.

Statistical analysis was performed with *Statistica* software. Comparisons between groups were performed using Chi Quadrat Test or independent Student’s *t*-test with *p* values < 0.05 considered statistically significant.

The ethical approval was not necessary because it was a retrospective study based on patients’ medical records.

## 3. Results

The characteristics are described in Table 1. There were no significant differences between the type of anastomosis in terms of age and sex. Adenocarcinoma G2 was the most frequent indication for right hemicolectomy procedure – 42% (30/72). The second most frequent indication was Crohn’s disease – 29% (21/72). The most often type of anastomosis performed was SSA – 35% (25/72) and the most rare 1 was SEA–15% (11/72). Among all of the procedures performed, 13 were urgent and 59 were elective. The urgent cases concerned 12 patients with Crohn’s disease and 1 patient with adenocarcinoma *G*2, all of whom suffered from mechanical obstruction. Operative results are presented in Table 2. (which presents the results for all the patients included in the study–group A) and 2.2 (which presents the results after excluding 13 patients whose procedures were urgent–group B). Total length of hospital stay (counted in days) was the longest after SEA (14, 26) and the shortest in SSA (12, 68) for group A but was the longest after ESA (13, 46) and the shortest after EEA (10, 88) for group B. Nevertheless the length of hospital stay after the operation was the longest in SEA in both groups (11, 15 and 9,63) and it was significantly higher compared to other types of anastomosis, in which the hospitalization time in group A did not exceed 10 days. Postoperative complications were noted in general in 24% (17/72) of the patients in group A and in 17% (10/59) in group B and are described in Table 3. They were the most frequent in SEA in both groups - 64% (7/11) in group A and 30% (3/10) in group B whereas in other types the rate of postoperative complications was significantly lower. The lowest rate was noted in SSA in group A–12% (3/25) and in EEA–13% (2/16) in group B. The rate of reoperation was the highest again in SEA in both groups–27% (3/11) in group A and 20% (2/10) in group B whereas no reoperations were noted in EEA and ESA. Total reoperation rate was 6% (4/72) in group A and 5% (3/59) in group B. Furthermore, the mortality rate did not differ significantly between studied groups, however was the highest in SEA in both groups – 18% (2/11) in group A and 20% (2/10) in group B. No deaths were noted in SSA and ESA.

**Table 1:** Patients’ clinicopathological characteristics.

	EEA (n = 21)	SSA (n = 25)	ESA (n = 15)	SEA (n = 11)
Age, yrs, mean (range)	63.29 (31–89)	61.15 (24–84)	62.69 (25–88)	63.46 (37–93)
Age, yrs, standard deviation	16.74	17.37	17.34	17.39
Gender M (n = 33) F (n = 39)	8 (24%)	10 (30%)	9 (28%)	6 (18%)
	13 (33%)	15 (39%)	6 (15%)	5 (13%)
Indication for surgery Adenocarcinoma *G*1 (n = 0)				
	0	0	0	0
Adenocarcinoma *G*2 (n = 30)	10	11	4	5
Adenocarcinoma *G*3 (n = 5)	0	2	1	2
Adenoma (n = 3)	0	1	2	0
Crohn’s disease (n = 21)	7	6	5	3
Necrosis (n = 2)	2	0	0	0
Mucinous carcinoma (n = 2)	1	1	0	0
NET (n = 8)	1	4	2	1
Medullar carcinoma (n = 1)	0	0	1	0

EEA = end-to-end anastomosis, ESA = end-to-side anastomosis, F = female, M = male, n = number, NET = neuroendocrine tumor, SEA = side-to-end anastomosis, SSA = side-to-side anastomosis.

**Table 2:** Operative results (for the whole study group) - group A.

	EEA (n = 21)	SSA (n = 25)	ESA (n = 15)	SEA (n = 11)	All patients	*p*
Total length of hospital stay (d), mean, median, range	12.72 10 4–33	12.68 9 5–20	12.74 9 5–44	14.26 10 5–36	12.77 11 4–44	.316294
Length of hospital stay after operation (d), mean, median, range	9.92 9 4–19	9.66 8 4–19	9.74 7 4–14	11.15 8 4–34	10.03 8 4–34	.074623
Postoperative complications	5 (24%)	3 (12%)	2 (13%)	7 (64%)	17 (24%)	.025385
Rate of reoperations	0 (0%)	1 (4%)	0 (0%)	3 (27%)	4 (6%)	.036661
Mortality rate	1 (5%)	0 (0%)	0 (0%)	2 (18%)	3 (4%)	.175234

d = days, EEA = end-to-end anastomosis, ESA = end-to-side anastomosis, SEA = side-to-end anastomosis, SSA = side-to-side anastomosis.

**Table 3:** Operative results (after excluding 13 patients whose procedures were urgent)–group B.

	EEA (n = 16)	SSA (n = 20)	ESA (n = 13)	SEA (n = 10)	All patients	*p*
Total length of hospital stay (d), mean, median, range	10.88 8 6–25	11.21 11 6–20	13.46 11 8–27	11.28 11 8–17	11.61 11 6–27	.410915
Length of hospital stay after operation (d), mean, median, range	8.76 7 5–19	9.42 9 5–19	9.02 9 7–14	9.63 9 5–16	9.21 8 5–19	.973122
Postoperative complications	2 (13%)	3 (15%)	2 (15%)	3 (30%)	10 (17%)	.679599
Rate of reoperations	0 (0%)	1 (5%)	0 (0%)	2 (20%)	3 (5%)	.327435
Mortality rate	0 (0%)	0 (0%)	0 (0%)	2 (20%)	2 (3%)	.177155

d = days, EEA = end-to-end anastomosis, ESA = end-to-side anastomosis, SEA = side-to-end anastomosis, SSA = side-to-side anastomosis.

The type of procedure - elective or urgent - did not significantly change the total length of hospital stay, the length of hospital stay after the operation and the mortality rate. However, it did affect the rate of postoperative complications and rate of reoperations. Nevertheless, after excluding urgent cases, SEA is still associated with higher occurrence of postoperative complications and reoperations however it is not statistically significant.

Regarding the frequency of different postoperative complications (Table 4)–wound infection was the most common complication, the highest in patients after EEA – 38% (8/21). Abdominal bleeding was noted only in patients after SEA – 45% (5/11). Anastomotic leak was only observed in patients following SSA – 20% (5/25). It was defined as the anastomotic insufficiency connected with the leak of intestinal content and the contrast out of the anastomosis and it was suspected based on the laboratory parameters indicating inflammation, bowel content or gas within the postoperative wound or drains and confirmed with a computed tomography scan of the abdomen performed with contrast. The complication was treated surgically and Hartmann’s procedure was performed. Bowel perforation was noted only in SEA patients – 27% (3/11).

**Table 4:** Postoperative complications (PC).

Postoperative complication (PC)	EEA (n = 21)	SSA (n = 25)	ESA (n = 15)	SEA (n = 11)
wound infection (n = 15)	8 (38%)	5 (20%)	0 (0%)	2 (18%)
wound eventration (n = 3)	3 (14%)	0 (0%)	0 (0%)	0 (0%)
abdominal bleeding (n = 5)	0 (0%)	0 (0%)	0 (0%)	5 (45%)
pulmonary embolism (n = 2)	0 (0%)	0 (0%)	0 (0%)	2 (18%)
pneumonia (n = 4)	0 (0%)	2 (8%)	2 (13%)	0 (0%)
anastomotic leak (n = 5)	0 (0%)	5 (20%)	0 (0%)	0 (0%)
bowel perforation (n = 3)	0 (0%)	0 (0%)	0 (0%)	3 (27%)

EEA = end-to-end anastomosis, ESA = end-to-side anastomosis, SEA = side-to-end anastomosis, SSA = side-to-side anastomosis.

## 4. Discussion

Despite many studies in the literature, the best type of anastomosis in right hemicolectomy for colon cancer remains an unresolved issue. In our study, the most frequent type of anastomosis was SSA (35%), followed by EEA (29%). According to study conducted by Liu Z et al, construction of an ileocolonic anastomosis with EEA for right hemicolectomy is recommended regarding significantly shorter operating time, lower incidence of complications including anastomotic leakage in comparison with ESA.^[15]^ A meta-analysis of randomized controlled trials regarding ileocolonic anastomoses published by Cohrane Collaboration in 2007, revealed that, if operation is performed for a colon cancer, EEA should be used as a recommended anastomosis technique following right hemicolectomy.^[16]^ On the other hand, our study revealed that EEA comes with high rate of postoperative wound infection and eventeration.

Howewer, Elod et al proved, that ESA implies significantly lower incidence of anastomotic leakage in comparison with EEA.^[4]^ Other studies also prove that ESA goes with favorable outcomes and is a safe procedure considering postoperative surgical complications hospital stay and guarantee the minimal risk of insufficiency and lethality.^[17–19]^ Likewise, results of our study prove the lowest rate of postoperative complications when ESA is performed.

In 2019, the Società Italiana di Chirurgia Endoscopica e Nuove Tecnologie prospective trial revealed that SSA isoperistaltic stapled intracorporeal anastomosis with hand-sewn enterotomy closure is the most frequently adopted technique to perform ileo-colic anastomosis after any indications for elective right hemicolectomy. The study also confirmed better short-term outcomes including shorter hospital stay and reduction of postoperative pain.^[20]^ Results of another study proved that in right colon tumors, SSA anastomotic technique should be used, being linked with the lowest chances of anastomotic leakage, also being considered with high probability a protective factor in the development of the fistula ^[4]^. On the contrary, our results show, that SSA is associated with higher occurrence of anastomotic leakage in comparison to other anastomotic types.

According to the literature, SEA develops the highest percentage of postoperative complications and should be considered as a significant risk factor of anastomotic leak after right hemicolectomy. This type of anastomosis is also connected with higher risk of fecal impaction.^[4,21]^ Our study confirms that SEA is associated with the highest number of postoperative complications, mortality and reoperations rate after right hemicolectomy. Rybakov et al confirmed that the only benefit of side-to-end anastomosis was a lower number of bowel movements.^[22]^

Finally, in our study only the open procedures were included in order to achieve a more homogenous group for the analysis. All anastomoses were handsewn, constructed in 2 layers of sutures using PDS 4-0 (the inner layer using non-interrupted suture and the outer using interrupted sutures). Despite of the mentioned above factors, we would like to say that we are aware of the limitations of the study which are the following: it is a single-center, retrospective study and has a small group of subjects including both patients undergoing surgery for a colonic cancer and Crohn’s disease as well as elective and urgent procedures. Therefore, further large, multicenter, randomized, controlled trials are required to confirm results of our study.

## 5. Conclusions

Taking into consideration all the sources mentioned above, there is no satisfying conclusion regarding the best type of anastomosis. The results of different studies are not consentaneous. A further multi-center randomized prospective controlled trial is needed in order to obtain a consensus and determine which anastomosis technique should be performed as for reduction of negative postoperative outcomes. Nevertheless, our study supplements the knowledge on this subject, because so far in the world literature there are few studies comparing different anastomotic configurations in the right hemicolectomy procedure, and the topic is extremely important clinically. Moreover, our research could inspire other researchers to explore this subject.

## Author contributions

**Conceptualization:** Beata Jabłońska, Sławomir Mrowiec.

**Data curation:** Joanna Machowicz.

**Formal analysis:** Joanna Machowicz, Maciej Wołkowski.

**Investigation:** Joanna Machowicz, Maciej Wołkowski.

**Methodology:** Joanna Machowicz, Maciej Wołkowski, Beata Jabłońska.

**Supervision:** Beata Jabłońska, Sławomir Mrowiec.

**Validation:** Beata Jabłońska.

**Visualization:** Maciej Wołkowski.

**Writing – original draft:** Joanna Machowicz, Maciej Wołkowski.

**Writing – review & editing:** Beata Jabłońska, Sławomir Mrowiec.
